# Weber’s Law as the emergent phenomenon of choices based on global inhibition

**DOI:** 10.3389/fnins.2025.1532069

**Published:** 2025-02-12

**Authors:** Marcin Penconek

**Affiliations:** Faculty of Psychology, University of Warsaw, Warsaw, Poland

**Keywords:** Weber’s Law, recurrent attractor network, choice circuit, global inhibition, emergence

## Abstract

Weber’s Law states that the ability to recognize the difference in intensity values is proportional to the reference intensity. The law is often generalized to the ratio principle which states that the proportionality also holds above the discrimination threshold. Experimental data showed that Weber’s Law fundamentally held in many sensory modalities including vision, audition, pressure, smell, and taste. However, violations were observed in many experimental studies and showed the mild convex relationship between stimulus intensities and Weber fractions. The magnitude of deviations from Weber’s Law was especially high in the low-intensity range in experiments on light brightness. The mechanistic foundation of Weber’s Law has recently received interest from neuroscience. It was postulated that the law constituted the emergent phenomenon arising in the choice circuit computing categorical choices based on global inhibition. This hypothesis suggested that the neurophysiological basis for Weber’s Law was linked to choice probabilities of a correct decision using linearly encoded stimulus intensities. Previous studies showed that the postulated mechanism led to the emergence of Weber’s Law. Our study showed that the same mechanism could also be responsible for the mild violation of Weber’s Law. The law approximately held for near-threshold discrimination, but did not hold as the ratio principle for easy discrimination with the high probability of a correct response. The revealed violation was qualitatively consistent with the experimental studies which showed the convexity of the relation between stimulus intensities and Weber fractions. However, the mechanism did not explain the magnitude of the deviations from Weber’s Law in the low-intensity range.

## Introduction

Weber’s Law is one of the best-documented phenomena in psychophysics. Early experiments showed that the ability to recognize the difference *ΔI* between two weights was proportional to the reference weight *I*, The just noticeable difference (*JND*) was proportional to stimulus intensity value, i.e., *JND* = *k*⋅*I*, where k was a constant. While the notion of the just noticeable difference describes the difference at a threshold, the principle can be extended by postulating that the difference *ΔI*(p) leading to choice probability p in favor of the higher value is proportional to reference intensity, i.e., *ΔI*(p) = *k*⋅*I*. We shall refer to this relationship as the ratio principle. The constant k is called the Weber fraction and it specifies the fractional increment of the value *I* necessary for observing the correct identification with probability p. The common interpretation of Weber’s Law was suggested by Fechner (1860, 1966). He postulated that the principle reflected the law of human perception and stated that the relation between the change of stimulus intensity in the physical world and its perceived change in the human mind was logarithmic (Fechner’s Law). He also proposed the generalized form of Weber’s Law: *ΔI*(p) = *k*⋅*I* + constant. Wundt, the father of psychology, suggested a different interpretation of Weber’s Law. He claimed that it reflected the law of judgment (Wundt, 1892; Marks, 1980) produced by a constant ratio relationship between compared sensations.

**FIGURE 1:**
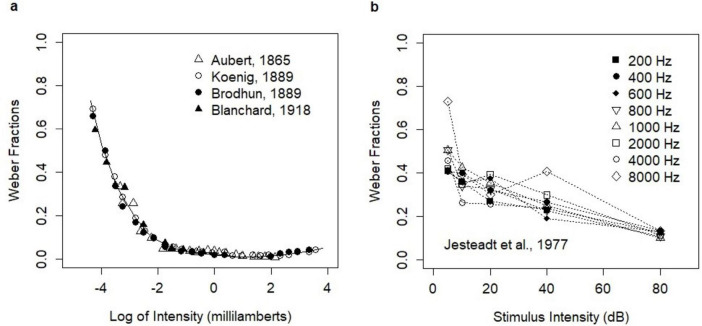
Empirical relationships between stimulus intensity and Weber fractions. **(A)** Early experiments on light brightness; the analysis of Hecht (1924). The figure was produced manually from the printed version of the paper (Hecht, 1924) with editorial changes. The print version is available in Open Access and the figure is reproduced here under the Creative Commons CC BY-NC-SA 4.0 License; https://creativecommons.org/licenses/by/4.0/legalcode. **(B)** The experiment of Jesteadt, Wier, and Green on the perception of pure tones (Table B-I). Reprinted with permission from Jesteadt et al. (1977). Copyright [1977], Acoustical Society of America. Note that the original paper presented the Weber fractions on the log scale.

Broad experimental evidence showed that Weber’s Law fundamentally held across many sensory modalities (Holway and Pratt, 1936) including vision, audition, pressure, smell, and taste. However, it was clear that the law could only hold within a reasonable stimulus intensity range. Indeed, violations of Weber’s Law for low stimulus intensities were reported in early experimental studies on light brightness (Aubert, 1865; König and Brodhun, 1889; Blanchard, 1918). In these studies, the ratio ΔI/I (Weber fraction) was higher in the low-intensity range. Moreover, the relation between stimulus intensities and the Weber fractions showed mild convexity across the entire stimulus intensity range. The experiments were summarized in the paper by Hecht (Hecht, 1924) who recalculated the original data of Aubert, Koenig, and Brodhun (Figure 1A). The deviation from Weber’s Law in the low-intensity range was further confirmed by many other studies (Von Helmholtz, 1866; Stiles and Crawford, 1933, 1934; Steinhardt, 1936; Blackwell, 1946; Aguilar and Stiles, 1954).

The convexity of the relation between stimulus intensities and the Weber fractions was also evidenced in the intensity discrimination of pure tones. Such relation was first reported by Riesz (1928) and then observed in many other experiments on the perception of pure tones (Dimmick and Olson, 1941; Schacknow and Raab, 1973; Penner et al., 1974). The relation was investigated further by McGill and Goldberg (1968) who coined the term “near miss” for the phenomenon. Rabinowitz et al. (1976) investigated the violations of Weber’s Law in 15 experimental studies on the loudness of 1000 Hz tone pulses. They concluded that the law held in the range from 10 to 40 dB, while it was violated below 10 dB (the efficiency of discrimination decreased) and in the range of 40–90 dB (the efficiency of discrimination steadily increased). The experimental study (Jesteadt et al., 1977) on the discrimination of pure tones of different frequencies showed a mild violation of Weber’s Law in the entire stimulus intensity range and for all frequencies. The revealed change in discrimination was described by the function △*I*/*I* = 0.463 ⋅ (*I*/*I*_0_)^−0.072^, where *I* was the intensity of tone and *I*_0_ was the intensity at the threshold. In this study, *ΔI* reflected an increment necessary to obtain the choice probability of 71% of correct responses. The study investigated stimulus intensities of 5–80 dB. In this range, the relation was convex and decreasing (Figure 1B).

Some experimental studies showed that the Weber fractions could increase in the high-intensity range. Such effects were reported in several experiments including the experiments on light brightness (König and Brodhun, 1889; Cobb, 1916; Holway and Pratt, 1936). The magnitude of these deviations was much smaller than the deviations in the low-intensity range and the effect was debated.

Experimental data on Weber’s Law usually does not involve the analysis of reaction time (RT). However, recent theoretical research suggested the link between Weber’s Law and the scale invariance of RT distributions (Simen et al., 2016; Pardo-Vazquez et al., 2019). The scale invariance of RT distributions postulates that RT distributions involving constant Weber fractions can be mapped to each other by rescaling time. In the first paper (Simen et al., 2016), the authors proposed a new unified theory of decision-making that explained Weber’s Law and predicted the scale invariance of RT distributions for decisions involving constant values of Weber fractions. This implied the invariance of the coefficient of variance (the ratio of standard deviation over mean) and the invariance of the skewness of RT distributions for such decisions. In the second paper (Pardo-Vazquez et al., 2019), the authors considered the constraint arising from the scale invariance (i.e., time-intensity equivalence in discrimination). They showed that under justifiable assumptions, it implied several features of the decision-making system including the linear relationship between the variance and the mean for encoding sensory evidence (as in the Poisson process), the power-law stimulus representation, and the perfect accumulation of evidence (as postulated by the drift-diffusion model; Ratcliff, 1978).

Weber’s Law has recently received interest in neuroscience. Experiments involving a sequential vibrotactile frequency discrimination task revealed that Weber fractions were relatively stable across a wide range of frequencies (20–200 Hz) for humans, comparable to Weber fractions for monkeys (in the range of 20–40 Hz), and hence Weber’s Law held (Mountcastle et al., 1990). Neuronal activity accompanying performing the task by monkeys was recorded in several studies (Mountcastle et al., 1990; Romo and Salinas, 2001, 2003; Hernandez et al., 2002; Romo et al., 2002, Romo et al., 2003, Romo et al., 2004). In particular, the experiment of Romo and colleagues (Romo et al., 2004) showed that the activity of neurons in the ventral premotor cortex (VPC) reflected response and memory for the first stimulus, response for the second stimulus, and their comparison which developed gradually. This study also suggested that the neurons encoding response were using the rate code which was linearly dependent on the stimulus frequency. This raises the question of how Weber’s Law is mechanistically implemented in the brain.

The hypothesis formulated by Deco and Rolls (2006) states that Weber’s Law is the emergent phenomenon linked to probabilistic choices based on global inhibition. Encoding of evidence is assumed to be linearly dependent on the stimulus intensity in the environment. The probability of a correct decision depends on the ratio of evidence due to divisive inhibition, which then is manifested as Weber’s Law. This hypothesis is conceptually close to the ideas of Wundt, as it postulates the role of judgment in eliciting choices based on the ratio of stimulus intensity levels.

This hypothesis was investigated from the computational perspective (Deco and Rolls, 2006; Deco et al., 2007) using a simplified version of Wang’s model (Wang, 2002). Wang’s model is a bio-physiologically realistic model of the choice circuit consisting of the recurrent attractor network of leaky integrate-and-fire neurons. The network is equipped with global inhibition producing categorical choices in two competing pools of excitatory neurons. Decision-making in this system involves the transition from a spontaneous state when neurons in the network fire with a low firing rate to a decision state when neurons in one of the two competing neuronal pools fire with a high firing rate. This pattern of the decision-making process is consistent with the neurophysiological experiments on decision-making [reviewed by Shadlen and Kiani (2013)]. The implementation of decision-making through the recurrent attractor network with global inhibition is considered to reflect the way categorical decisions are implemented in the brain (Rolls, 2013). In the studies investigating Weber’s Law using Wang’s model (Deco and Rolls, 2006; Deco et al., 2007) the authors showed that the constant Weber fractions emerged as a result of comparisons produced by the model assuming the linear relationship between stimulus intensity and model inputs. The model produced choice probabilities based on the ratio of model inputs thus leading to Weber’s Law. The analysis also showed that Weber’s Law held in the multi-stable regime (i.e., when the spontaneous state was stable) and it broke in the bi-stable regime (i.e., when the spontaneous state was unstable). The studies were conducted in the context of the vibrotactile frequency discrimination task with the multi-stable regime applicable to the frequency range of 20–40 Hz and the bi-stable regime applicable to the frequency above 40 Hz (40–260 Hz). These stimulus ranges reflected the ranges investigated experimentally.

However, the analysis showed no convexity of the relation between stimulus intensities and the Weber fractions. In this paper, we show that the mechanism suggested by the hypothesis can also explain the convexity. We also analyze the scale invariance of RT distributions. Modeled RT distributions showed scale invariance proximity, but they violated the property in the strict sense. The analyses were conducted using an alternative implementation of the choice circuit based on the recurrent attractor network of binary neurons equipped with a global inhibition mechanism (Penconek, 2020).

## Materials and methods

### Decision-making model

The choice circuit consists of the pool of *N* = 1000 excitatory neurons. The role of inhibitory neurons in providing network stability and facilitating winner-take-all competition is ensured by the global inhibition mechanism implemented in the update rule. This way of implementing inhibition is motivated by the mean-field analysis of excitatory-inhibitory (E-I) networks. It assumes that the inhibitory pool is at equilibrium through the separation of time scales. Such an idea is often used in modeling practice (e.g., Wong and Wang, 2006; Gerstner et al., 2014). Global inhibition is postulated as one of the key principles of the architecture of the choice circuit (Rolls, 2013) and assumes that all excitatory neurons in the circuit receive the same (time-variable) level of inhibition. The global inhibition mechanism implemented in the update rule replaces the pool of inhibitory neurons which is not directly modeled.

Excitatory neurons are represented by the binary state function s_*t*_(i) which assumes value 1 if neuron *i* is in the effective refractory period and 0 otherwise. An action potential is emitted at the time of updating the neuron into state 1. During the refractory period, the neuron cannot emit a new spike. The length of the effective refractory period equals *d* = 1/0.07 which sets the maximum frequency at which neurons can fire action potentials (i.e., 70 Hz). The effective refractory period is also the neuronal integration time. In this convention, the summary excitatory input to neuron *j* from all its presynaptic neurons can be written as the sum of their states:


$$\int_{t-d}^{t}\sum_{i=1}^{N}\delta \,\left(\tau -t_{k}\,\left(i\right)\right)\,w_{i\,j}\,d\,\tau =\sum_{i=1}^{N}\;s_{t}\,\left(\text{i}\right)\,w_{i\,j}$$

where *t*_*k*_ (*i*), *k* = 1, 2 is the sequence of times at which spikes occurred in the presynaptic neurons *i* and *w*_*ij*_ are the presynaptic connections of the neuron *j*. The integration is taken over the period (t-d, t) where τ is the integration time variable and δ is the Dirac delta function. Connections are random and binary that is *w*_*ij*_ = 0 or  1. No restrictions on the connections are imposed. In particular, the network is not assumed to be symmetric. The resulting summary excitatory postsynaptic potential (EPSP) is assumed to be normalized by the inverse of the number of presynaptic connections.

Network inhibition is implemented through the global inhibition mechanism which is the quadratic function of the average activity of the network of excitatory neurons parameterized with the inhibitory constant Θ. Thus, the update rule for neuron *j* can be written as:


$$s_{t}\,\left(\text{j}\right)=1\,\mathrm{iff}\;\frac{\sum_{i=1}^{N}\;s_{t}\,\left(\text{i}\right)\,w_{i\,j}}{\sum_{i=1}^{N}w_{i\,j}}=\frac{1}{\Theta }\cdot {\left(\frac{\sum_{i=1}^{N}\;s_{t}\,\left(\text{i}\right)}{N}\right)}^{2}$$

where the value of the left-hand side of the update rule reflects the summary excitatory postsynaptic potential (EPSP), and the right-hand side – the sum of the summary inhibitory postsynaptic potential (IPSP) plus the activation threshold (i.e., the difference between the threshold potential and the resting potential). The proposed update rule ensures a self-sustained activity of the network with no exogenous stochastic stimulation, provided the initial state of the network is non-zero. The inhibitory constant Θ determines the equilibrium level of neuronal excitation at which the summary EPSP (expressed by the left-hand side of the update rule) equals the summary IPSP plus the activation threshold (expressed by the right-hand side). The choice of Θ = 0.13 ensures self-sustained spiking activity with the population-average total network firing rate of 9.24 Hz ( ± 0.09). The network is initialized with random non-zero states with probability Θ.

The network follows continuous evolution with asynchronous updates governed by the Poisson point process with the rate 0.006, except for constant refractory periods. i.e., updates for active neurons follow their respective refractory periods. The Poisson process governing updates for inactive neurons is assumed to reflect the physical process of subthreshold fluctuations of the electrical potential leading to the emission of a spike. The choice of the Poisson process is consistent with previous modeling practice (Dayan and Abbott, 2005; Wójcik, 2018).

**FIGURE 2:**
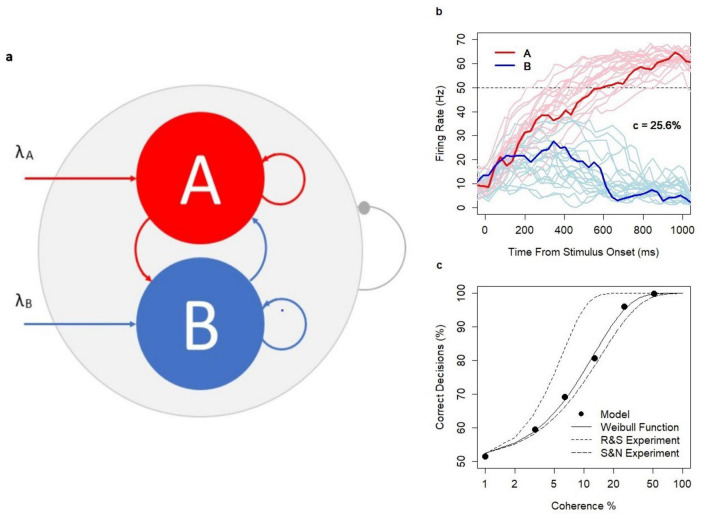
Choice Circuit. **(A)** The schematic representation of the choice circuit architecture: the recurrent network of randomly connected excitatory neurons with decision pools A and B receiving parallel inputs. The system is equipped with the global inhibition mechanism (shown as the line ending with a dot) facilitating self-sustained neuronal activity and winner-take-all competition between decision pools. **(B)** Firing rate in pools A and B during decision formation for model runs with coherence level *c* = 25.6% and the threshold value 50 Hz indicated by the dotted line. Thicker lines show a single model run selected from a wider range of model runs indicated by thinner lines. **(C)** Psychometric function: *N* = 12,000 model runs (2000 per coherence level *c* = 0, 3.2, 6.4, 12.8, 25.6, 51.2%, and decision threshold 52.5 Hz), SEM: 0.09–1.14; dotted lines: approximation of the experimental data of Shadlen and Newsome (S&N) and Roitman and Shadlen (R&S) with the Weibull function; solid line: approximation of model estimates: α = 12.5 and β = 1.18. The analysis was previously featured in the paper (Penconek, 2022) and is reproduced under the Creative Commons Attribution 4.0 International License; https://creativecommons.org/licenses/by/4.0/legalcode.

The network of excitatory neurons consists of three pools: two decision pools, A and B (each *n* = 100), with a higher density d_1_ = 0.55 of random connections within each set and the rest of the network with the density of random connections d_2_ = 0.36. The architecture of the choice circuit is summarized in Figure 2A. The higher density of connections in the decision pools is assumed to be a result of Hebbian learning (Hebb, 1949); no learning mechanism is introduced in the model. A new set of connections is generated before each model simulation and is kept constant during the simulation.

The network works in the multi-stable regime, i.e., the spontaneous state, and both decision states A and B are semi-stable. The spontaneous state is characterized by a low firing rate of neurons in the decision pools (mean firing rate in the decision pool: 10.3 Hz, ± 5.2). Decision states are characterized by a high firing rate in the winning decision pool (mean: 59.7 Hz, ± 4.7) and by the suppressed spiking activity in the losing pool (mean: 2 Hz, ± 1.4). The firing rate of 50 Hz reflects the lower border of the decision state and hence is the default decision threshold level. The firing rate is calculated based on 30 ms lags sampled each 10 ms.

The decision pools A and B are selective to inputs. Inputs are provided in parallel to 50% of neurons in A and B, respectively. Stimuli are encoded in the time-variable sequences of excitatory spikes which modify the left-hand side of the update rule. The values of the sequences are sampled each 30 ms from the Poisson distribution (Poisson inputs) with parameters λ_*A*_ and λ_*B*_ reflecting the strength of the evidence in favor of decisions A and B, respectively. When inputs are provided, the system facilitates the neuronal integration process from the spontaneous state (starting point of all simulations) to one of the decision states, respectively. The range of integration time (in hundreds of milliseconds) is consistent with experimental evidence (Shadlen and Newsome, 2001; Roitman and Shadlen, 2002). Several examples of the decision process (in the context of dots motion experiments with coherence *c* = 25.6%) were illustrated in Figure 2B.

The model has several control parameters: the size of decision pools A, B (*n* = 100 each) and the rest of the network (800), the inhibition constant (θ = 0.13), parameters controlling the time evolution of the network (*d* = 1/0.07 and the rate for inactive neurons 0.006), the density constants (d_1_ = 0.55 and d_2_ = 0.36), and the decision threshold (default value 50 Hz). Some model parameters are arbitrary (network size and sizes of the decision pools A and B). Other parameters are inspired by empirical evidence, e.g., *d* = 1/0.07 defines the maximum firing rate of neurons (70 Hz). This value is consistent with the maximum firing rate of LIP neurons implicated in performing the evidence accumulation process in the brain. The rate for inactive neurons (0.006) controls the speed of the integration process within the pool of neurons and was set by computational experiments. The density constants d_1_ and d_2_ are set to control the emergent dynamic properties of the model (i.e., multi-stability; Penconek, 2020). In the analyses presented in this paper, the control parameters are fixed at their default values.

Research evidence (Shadlen and Newsome, 2001; Roitman and Shadlen, 2002) showed that in the dots motion discrimination experiments, the relationship between coherence level c (*c* = 0, 3.2%, 6.4%, 12.8%, 25.6%, and 51.2%) defining the difficulty of the task and the probability of a correct response followed the Weibull psychometric function. Model predictions were consistent with the experimental data. Decision accuracy followed the psychometric function with realistic parameters α = 12.5, β = 1.18 (Figure 2C). As in the experiments, reaction times (RTs) increased with task difficulty, were shorter for correct decisions, and longer for erroneous decisions across all coherence levels (*c* > 0). The model was previously used to investigate the alternative neuronal mechanisms of speed-accuracy tradeoff (Penconek, 2022).

The model considered in this paper is conceptually similar to the mechanistic model proposed by Wang (Wang, 2002). Both models feature two neuronal pools A and B with stronger connectivity within each pool and are equipped with global inhibition which facilitates winner-take-all competition between the pools. Such an organization is consistent with the contemporary view of how decision-making is mechanistically implemented in the brain (Rolls, 2013). However, the models differ in several respects: the level of detail in which neurons in the network are represented (binary neurons in this model vs. leaky integrate-and-fire neurons in the Wang model), random connectivity (all or none connections in this model vs. weighted connections in the Wang model), and the way, in which the global inhibition is implemented (the global inhibition mechanism in this model vs. common pool of inhibitory neurons in the Wang model). Some dynamic properties of the models are also different. The model considered here exhibits self-sustained network activity while the Wang model requires external stimulation for such an activity.

### Analysis of Weber’s Law

The analysis of Weber’s Law involved eliciting the stimulus intensity value λ_*B*_ (λ_*B*_ > λ_*A*_) for which the probability of correctly identifying the higher value (convergence to decision B) is equal to the assumed choice probability p. The analysis was conducted for 10 stimulus intensity values *λ_*A*_* = 2, 3, …, 11, and three choice probability values *p* = 0.85, 0.75, and 2/3. Choice probability 0.85 was used in the previous research using Wang’s model (Deco and Rolls, 2006; Deco et al., 2007); probability 0.75 was commonly used in psychological experiments. The procedure involved generating *N* = 5000 model simulations for each stimulus intensity level *λ_*A*_* (in total *N* = 50,000 simulations) and randomly chosen (uniform distribution) value of stimulus intensity *λ_*B*_* in the interval (*λ_*A*_*, 2⋅*λ_*A*_*). Model simulations were conducted with the standard values of the control parameters and the decision threshold of 50 Hz. Stimulus was delivered after 500 ms of spontaneous model run and was continued for the period of 5000 ms.

Estimates were based on the Weibull psychometric function:


$$\%correct=1-\frac{1}{2}\exp\left(-{\left(\frac{c}{\alpha }\right)}^{\beta }\right)$$

were c = (*λ_*B*_* – *λ_*A*_*)/(*λ_*A*_* + *λ_*B*_*). The choice of the Weibull psychometric function was motivated by experimental results (Shadlen and Newsome, 2001; Roitman and Shadlen, 2002; Churchland et al., 2008) and reflected the relation between coherence and choice probability in the model (Penconek, 2022). The estimation procedure involved fitting the parameters α and β of the Weibull psychometric function, using the inverse function to estimate the coherence level reflecting the assumed choice probability p, and calculating the value *λ_*B*_* corresponding to this coherence level. Confidence intervals (95% CI) were estimated with 1,000 bootstraps using the standard bootstrap procedure involving selecting subsamples with replacement. The estimated *λ_*B*_* values were used to calculate the difference *Δλ*(p) and Weber fractions *Δλ*(p)/ *λ_*A*_*.

**FIGURE 3:**
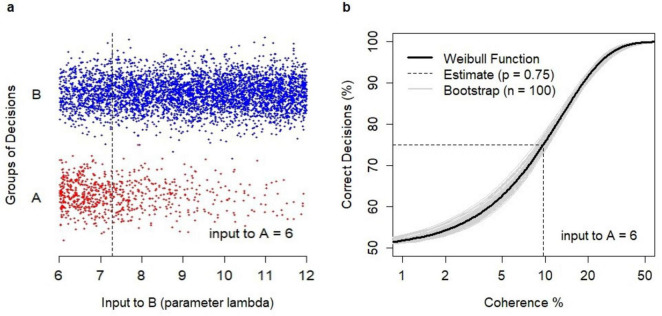
Analysis of binary choices. **(A)** Dataset of *N* = 5000 binary choices generated by the model for λ_A_ = 6 and random values of λ_B_ (λ_B_ > λ_A_). Correct decisions (B, coded as –1) were shown as blue dots; erroneous decisions (A, coded as +1) were shown as red dots. The data points were jittered vertically to avoid cluttering. The obtained estimate of λ_B_ = 7.29 (dotted line) for the choice probability *p* = 0.75. **(B)** Estimation of the relationship between coherence and the percentage of a correct decision using the Weibull psychometric function. The revealed estimate of coherence level *c* = 9.68% at which the probability *p* = 0.75 was obtained (dotted line). A sample of bootstrap estimates (*n* = 100) of the relationship (gray lines).

An example is shown in Figure 3. Model simulations generated binary choices (Figure 3A) for varying values of *λ_*B*_*, where *λ_*B*_* was the parameter of the Poisson sequence defining the time-variable inputs to B, and reflecting the strength of evidence in favor of B. Decisions were grouped into correct ones (i.e., decisions B) and erroneous ones (i.e., A). The estimation of the relationship between coherence and the probability of a correct decision followed the Weibull function (Figure 3B) with parameters α = 12.8, β = 1.31. The probability *p* = 0.75 was obtained for the coherence value of 9.68% which reflected *λ_*B*_* = 7.29 (plotted as the dotted line in Figure 3A).

### Analysis of reaction times (RTs) and scale invariance

The analysis required generating a separate set of *N* = 2000 simulations for each pair of *λ_*A*_* and *λ_*B*_* parameters, where the parameters *λ_*B*_* were the estimates from the previous analysis for the choice probability *p* = 0.75 (in total *N* = 20,000 simulations). These sets of simulations were used to validate the probability from the previous analysis and allowed us to analyze the distributions of RTs. Model simulations were conducted with the standard values of the control parameters and the decision threshold of 50 Hz. Stimulus was delivered after 500 ms of spontaneous model run and was continued for the period of 5000 ms. No non-decision time was assumed.

Scale invariance of RT distributions was tested by rescaling the modeled RT distributions to the reference RT distribution for the stimulus level *λ_*A*_* = 9. First, the RT distributions were scaled by the inverse of their medians. Then, the correcting scaling parameter was chosen to minimize the Kolmogorov-Smirnov (K-S) statistic (i.e., to minimize the probability of rejecting the null hypothesis that the rescaled distributions are the same). The correcting scaling parameter was in the range from 0.9703 (for stimulus level *λ_*A*_* = 2) to 1.0060 (for stimulus level *λ_*A*_* = 11). The reference RT distribution (for *λ_*A*_* = 9) was chosen to minimize the sum of K-S statistics across all stimulus levels.

In the analysis of RT skewness and when plotting modeled RT distributions on the Cullen and Frey graph (Cullen and Frey, 1999), we used the following formulas for skewness and Pearson’s kurtosis:


$$\hat{s\,k}\,\left(x\right)=\frac{\sqrt{n\,\left(n-1\right)}}{n-2}\cdot \frac{\frac{1}{n}\,\sum_{i}{\left(x_{i}-\bar{x}\right)}^{3}}{{\left(\frac{1}{n}\,\sum_{i}{\left(x_{i}-\bar{x}\right)}^{2}\right)}^{3/2}}$$

$$\hat{k\,r}\left(x\right)=\frac{n-1}{\left(n-2\right)\,\left(n-3\right)}\cdot (\left(n+1\right)\cdot$$

$$\frac{\frac{1}{n}\,\sum_{i}{\left(x_{i}-\bar{x}\right)}^{4}}{{\left(\frac{1}{n}\,\sum_{i}{\left(x_{i}-\bar{x}\right)}^{2}\right)}^{2}}-3\left(n-1\right))+3$$

The above formulas provide the unbiased estimators of skewness and kurtosis from a sample of n independent, identically distributed observations x_*i*_ (Casella and Berger, 2002). These estimators are implemented in R to calculate skewness and kurtosis in Cullen and Frey graph (Delignette-Muller and Dutang, 2015). It is clear that the above estimators of skewness and kurtosis are scale-invariant, i.e., $\hat{s\,k}\,\left(c\,x\right)=\hat{s\,k}\,\left(x\right)$ and $\hat{k\,r}\,\left(c\,x\right)=\hat{k\,r}\,\left(x\right)$ for c > 0.

## Results

Weber’s Law postulates that JND is proportional to the reference intensity. In modern experimental studies, the relation is assumed to hold for any choice probability. For choice probability p, *ΔI*(p) is defined as the difference between stimulus intensities *I*_*B*_ and *I*_*A*_ (*I*_*B*_ > *I*_*A*_), i.e.,


$$\Delta \,I\,\left(p\right)=I_{B}-I_{A}$$

such that the probability of correctly identifying the higher value of stimulus *I*_*B*_ when comparing it to *I*_*A*_ equals p. The ratio principle states that for all values of *I*_*A*_, the ratio of *ΔI*(p) over *I*_*A*_ is constant i.e.,


$$\frac{\Delta \,I\,\left(p\right)}{I_{A}}=k$$

where *k* is the Weber fraction. Generalized Weber’s Law is expressed as a linear relationship between *I*_*A*_ and *ΔI*(p).

### Assumptions about encoding

Our hypothesis assumes that stimulus intensity is linearly encoded in the stochastic spiking activity of neurons. In the model considered here, the input level is determined by the parameter λ defining the stream of Poisson spikes delivered to the system. Thus, the assumption of linear encoding states that λ = a⋅*I* + b for some *a* > 0, and b. Although a positive value of b associated with background network noise is biologically plausible, it increases the Weber fractions. The inverse of the Weber fraction represents the efficiency of discrimination. The fitness maximization principle implies that the efficiency of discrimination should be maximized in biological systems, i.e., the Weber fractions should be minimized which holds for *b* = 0. This justifies the assumption that stochastic spiking activity is proportional to stimulus intensity, i.e., λ = a⋅*I* for some *a* > 0. The relation enables us to express the Weber fractions in units reflecting input levels λ to the system. Indeed, under the above assumptions:


$$\frac{\Delta \,I\,\left(p\right)}{I_{A}}=\frac{I_{B}-I_{A}}{I_{A}}=\frac{\lambda_{B}-\lambda_{A}}{\lambda_{A}}$$

Hence, we can identify stimulus intensity *I* with the input parameter λ. In other words, we can express the quantity from the environment in the human-centric units reflecting the neuronal activity in the brain.

### Proportional encoding and the emergence of Weber’s Law

**FIGURE 4:**
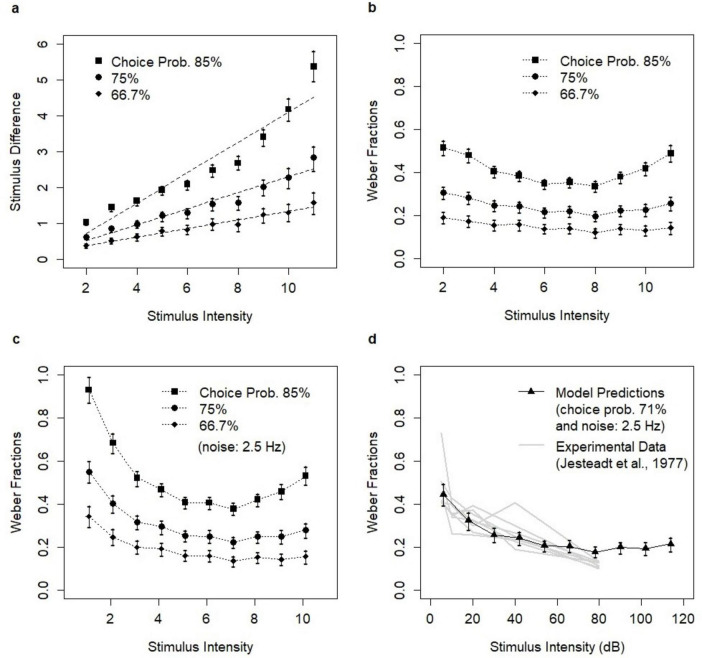
Model predictions. **(A)** Stimulus difference as the function of stimulus intensity for three assumed choice probabilities *p* = 0.85, 0.75, and 2/3. The relationships were approximated by the linear functions (dotted lines). **(B)** Revealed Weber fractions as the function of stimulus intensity for choice probabilities *p* = 0.85, 0.75, and 2/3. **(C)** Revealed Weber fractions assuming independent background noise of 25 neurons delivering inputs, each spiking at 2.5 Hz. **(D)** Fit between the experimental data (Jesteadt et al., 1977) and model predictions for choice probability *p* = 0.71 (as in the experiment) and background noise of 2.5 Hz. Each estimate involved *N* = 5,000 model simulations. Bars represent confidence intervals (95% CI) which were based on the standard bootstrap procedure involving drawing 1,000 subsamples with replacement.

In the following analysis, we assumed that the encoding of stimulus intensity was proportional to its value in the environment. The analysis was conducted for three choice probabilities *p* = 2/3, 0.75, and 0.85 and 10 stimulus intensity levels λ_*A*_ = 2, 3, … 11. For each stimulus level λ_*A*_ and probability p, the analysis involved estimating the value of λ_*B*_ (λ_*B*_ > λ_*A*_) such that the probability of correctly identifying λ_*B*_ as the higher value when comparing it to λ_*A*_ equaled p. The estimated values λ_*B*_ were then used to calculate stimulus differences and Weber fractions. The analysis revealed that the difference increased as a function of stimulus intensity and that this relationship was approximately linear for the choice probabilities *p* = 2/3 and 0.75 (Figure 4A). The linear relationship was consistent with the generalized form of Weber’s Law: *ΔI*(p) = *k*⋅*I* + constant. The linearity broke for the choice probability *p* = 0.85 (Figure 2A) suggesting that the ratio principle did not hold for high values of choice probability p, i.e., for easy discrimination.

### Violation of Weber’s Law as the ratio principle

The relationships between stimulus intensity and Weber fractions systematically deviated from Weber’s Law over the entire range of stimulus levels (Figure 4B). The revealed relations were apparently convex, decreasing in the low to medium range and increasing for high stimulus intensity levels. The violation of Weber’s Law was particularly evident for *p* = 0.85 and was also observed for *p* = 0.75. This conclusion was supported by fitting the quadratic (parabolic) function using the linear regression which produced positive second-order parameters (*t*-value = 4.432, *p*-value = 0.00304, and *t*-value = 7.882, *p*-value = 1e^–4^ for choice probabilities 0.75 and 0.85, respectively). The convexity was not confirmed for *p* = 2/3 (*t*-value = 2.341, *p*-value = 0.0518). The apparent convexity was linked to finite size effects; the ratio-based efficiency of discrimination was lower for low stimulus levels, reached its maximum at medium levels (the lowest values of Weber fractions), and was again lower (due to saturation) at high levels. The convexity increased as a function of choice probability and hence, the non-linearity of the relation might be hardly noticeable for low choice probability values (*p* ≤ 2/3), i.e., when Weber’s Law is considered at thresholds, as originally proposed by Weber.

### Impact of the background network noise

The above analyses assumed that encoding was proportional to stimulus intensity. The assumption was justified by the fitness maximization principle. However, experimental evidence showed that neurons in the networks consisting of excitatory and inhibitory neurons exhibit spontaneous spiking activity at the level of a few spikes per second. This suggested the existence of the background network noise in the form of an independent homogenous Poisson process. Such background noise created no effect on the revealed stimulus difference Δ*I* beyond shifting the stimulus value. However, it did affect the revealed values of Weber fractions. Indeed, in the presence of noise at the level of λ_0_ (λ_0_ > 0), the Weber fraction (in human-centric units) equaled:


$$\frac{\Delta \,I\,\left(p\right)}{I_{A}}=\frac{I_{B}-I_{A}}{I_{A}}=\frac{\lambda_{B}-\lambda_{A}}{\lambda_{A}-\lambda_{0}}$$

The level of the noise was determined by two factors: the spiking activity of a neuron (in Hz) and the number of neurons projecting inputs to the decision pool. This number should be large enough to deliver time-varying random Poisson inputs with mean value (and variance) λ in the range 10–15. We assumed that the inputs were delivered by 25 neurons with the activity of 2.5 Hz. The presence of noise affected the revealed relationships in a non-linear way, increasing Weber fractions and producing further convexity of the relation between stimulus intensity and Weber fractions (Figure 4C). The increase of Weber fractions was stronger in the low-intensity range. The mechanism discussed in this section could be considered the “how-possibly” explanation in the sense suggested by Piccinini (2020).

### Qualitative consistency of model predictions with experimental data

While the results showed the violation of Weber’s Law as the ratio principle, the form of the violation was consistent with experimental studies. The convex relation between stimulus intensity and Weber fractions was reported in many experimental studies on light brightness in which the deviations from Weber’s Law were observed [e.g., Aubert, 1865; König and Brodhun, 1889; Blanchard, 1918, reported by Hecht (1924), see Figure 1A]. Such deviations were also consistent with a wide range of experimental results on discrimination of pure tones (Riesz, 1928; Dimmick and Olson, 1941; McGill and Goldberg, 1968; Schacknow and Raab, 1973; Penner et al., 1974; Rabinowitz et al., 1976). The convex relation was postulated by the function describing the discrimination of pure tones (Jesteadt et al., 1977). The study investigated Weber fractions for choice probability 0.71. The intensity of pure tones covered the range of 5–80 dB. Model predictions were quantitatively consistent with the experimental data (Figure 4D) assuming the noisy spiking at 2.5 Hz. Mapping between the range of stimulus intensities (in human-centric units) and intensities of pure tones (in dB) was arbitrary. The choice of the range was informed by the limits of the human auditory system.

Background network noise may contribute to the violation of Weber’s law in the low-intensity range, but it was unlikely to explain the effects observed in early experiments on light brightness [ König and Brodhun, 1889; Blanchard, 1918; reported by Hecht (1924), see Figure 1A]. The lowest reported Weber fractions in these experiments were at the level of ∼0.01, whereas the highest values were 70-fold higher, at the level of ∼0.7. We hypothesize that the observed violation in the low-intensity range was related to a different neuronal mechanism. Investigating such a mechanism was beyond the scope of the current paper.

### Analysis of reaction times (RTs)

In this section, we analyze reaction times (RTs) for decisions involving the discrimination between *λ_*A*_* and *λ_*B*_*, where *λ_*B*_* is the estimate of stimulus intensity value that produces the pre-defined choice probability *p* = 0.75. Reaction times are not usually reported in experimental studies on Weber’s Law. However, the analysis of reaction times is interesting due to the theoretically postulated scale invariance of RT distributions (Simen et al., 2016; Pardo-Vazquez et al., 2019). Such property can be informative of the underlying mechanism of Weber’s Law.

**FIGURE 5:**
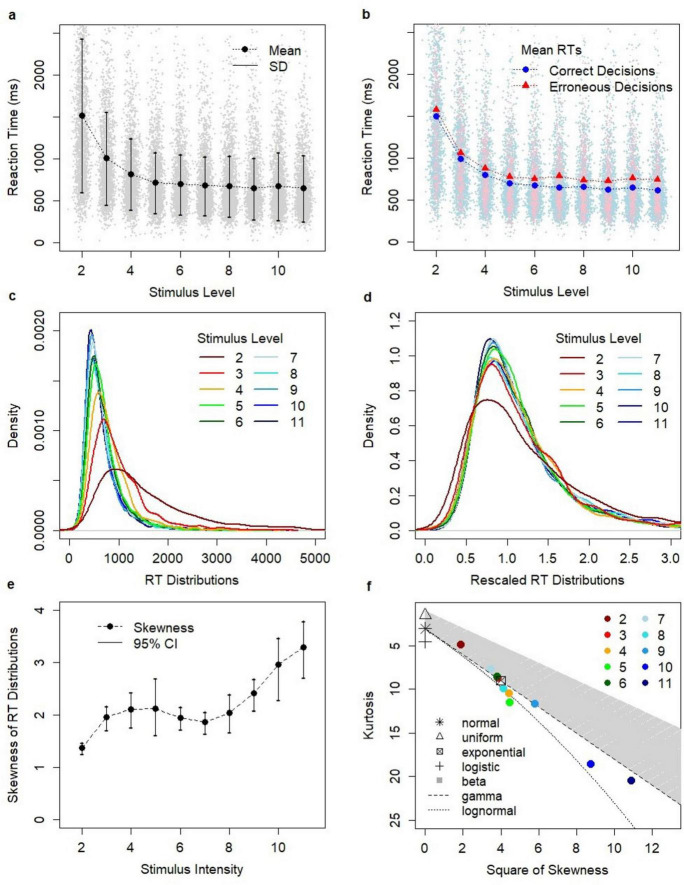
Reaction times (RTs). **(A)** The relation between stimulus intensity and mean RTs. Bars represent standard deviation (SD). **(B)** Mean RTs for correct (blue marks) and erroneous (red marks) decisions. **(C)** RT distributions for different stimulus levels: 2, 3,… 11. **(D)** Rescaled RT distributions. Rescaling based on the minimization of K-S statistics. **(E)** The relation between intensity levels and RT skewness. Bars represent 95% CI based on 10,000 bootstraps. **(F)** Cullen and Frey graph for the modeled RT distributions.

Reaction times were longer for low stimulus levels (Figure 5A). Erroneous responses were slower than correct responses across the entire range of stimulus values (Figure 5B). The mean difference between RTs on erroneous and correct responses was positive and was ranging from 75 ms (stimulus level *λ_*A*_* = 3) to 139 ms (stimulus level *λ_*A*_* = 7). This pattern of RTs was consistent with the previous analysis based on the model which showed the positive difference between RTs on erroneous and correct responses, i.e., the default RT pattern, for the decision threshold level of 50 Hz (Penconek, 2022). The modeled RT distributions were skewed (Figure 5C) which was consistent with empirical evidence.

### Scale invariance proximity

The scale invariance of RT distributions postulates that RT distributions involving constant Weber fractions can be mapped to each other by rescaling time. The rescaled RT distributions were plotted in Figure 5D. All RT distributions were rescaled to fit the reference RT distribution for *λ_*A*_* = 9. This involved rescaling by median and applying the correcting factor to minimize the Kolmogorov-Smirnov statistic. The analysis suggested that the scale invariance held for all stimulus levels except its lowest value *λ_*A*_* = 2 (Kolmogorov-Smirnov test, *D* = 0.085418, *p* = 1.089e^–6^). However, the Kolmogorov-Smirnov test is based on the supremum norm and is not sensitive to minor (but systematic) differences in the tails of tested distributions; conversely, descriptive statistics such as distribution skewness and kurtosis are.

The analysis of skewness showed that the skewness of modeled RT distributions increased in a non-linear way as a function of stimulus intensity (Figure 5E). Confidence intervals (confidence level 95%) were obtained by bootstrap estimates of skewness (the standard bootstrap procedure involving drawing 10,000 subsamples with replacement). Note that while the mean and the variance are affected by rescaling, the skewness of distribution is not. Thus, the observed differences in skewness of RT distributions revealed the violation of scale invariance. These differences were also shown in the Cullen and Frey plot (Figure 5F). The Cullen and Frey graph (Cullen and Frey, 1999) plots the squared skewness versus kurtosis of the observed distributions relative to selected theoretical distributions. This analysis suggested that the RT distributions could be approximated by the family of gamma distributions. Gamma distributions are parameterized with two parameters shape α and rate β (or alternatively, by shape and scale). Gamma distributions are interesting in the context of scale invariance, because the postulated scale invariance implies the same value of the shape parameter α. Conversely, the skewness of gamma distributions depends only on their shape and is given by $2/\sqrt{\alpha }$. Thus, different skewness of compared RT distributions implies different values of the shape parameter α and a violation of scale invariance.

The analysis of RT distributions showed that the scale invariance of RT distributions was violated in model simulations. The violation of scale invariance was not surprising when considering constraints of the neuronal network such as the maximum firing rate of neurons in the network. This constraint is not scale invariant. Such constraint was implemented in the model (maximum firing rate was equal to 70 Hz) and is also present in the biological networks. In neurophysiological experiments, the maximum firing rate of biological neurons during the neuronal integration process was in the range of 65–70 Hz. This range was observed in the LIP area of the brain implicated for decision-making in the dots motion discrimination tasks (Shadlen and Newsome, 2001; Roitman and Shadlen, 2002; Churchland et al., 2008).

## Discussion

Weber’s experiments on the discrimination of weights revealed that the just noticeable difference (*JND*) was proportional to the reference stimulus intensity. These experiments were based on two-choice decision-making tasks which involved two information processing stages: stimulus encoding for alternative options and the comparison (choice) between alternative options. The widely accepted interpretation of Fechner (1860, 1966) postulated that the principle reflected the law of human perception: the relation between the change of stimulus intensity in the physical world and its perceived change in the human mind was logarithmic (Fechner’s Law). Such interpretation required an additional assumption that the perceived change in the mind was proportional to the revealed value of *JND*. An alternative interpretation of Weber’s Law was suggested by Wundt who considered the relationship as the law of judgment (Wundt, 1892; Marks, 1980). Wundt noticed that revealing subjective sensations required judgments such as decision-making. He also formulated the law of relativity which stated that *JND* corresponded to a constant ratio relationship between compared sensations (Wundt, 1892; Marks, 1980).

In this paper, we investigated the hypothesis that Weber’s Law in the form of the ratio principle is the emergent phenomenon linked to probabilistic choices based on global inhibition. The probability of a correct decision depended on the ratio of stimulus intensities, thus, leading to Weber’s Law (Deco and Rolls, 2006). The hypothesis was previously investigated using Wang’s model (Deco and Rolls, 2006; Deco et al., 2007). These studies showed that indeed, the choice circuit equipped with global inhibition produced choices based on the ratio of intensity values (when it operated in the multi-stable regime) and supported the proposed mechanism behind Weber’s Law. However, many experimental studies showed the violation of Weber’s Law and revealed the mild convex relationship between stimulus intensity and Weber fractions. We explained these phenomena by the same neuronal mechanism.

Our results showed that the relation between stimulus difference and the reference intensity could be approximated by the linear relationship near the discrimination threshold, as originally proposed by Weber. However, the linear relation broke for easy discrimination when probability of identifying a higher value was high. Hence, the ratio principle did not hold. Our analysis showed a mild violation of the ratio principle across the entire range of stimulus intensity levels which formed the convex relationship between stimulus intensities and Weber fractions, as observed experimentally. Such violations of Weber’s Law in the entire stimulus intensity range were observed in many experimental studies including historical experiments on light brightness (Aubert, 1865; König and Brodhun, 1889; Blanchard, 1918; see Hecht, 1924) and experiments on discrimination of pure tones (Riesz, 1928; Dimmick and Olson, 1941; McGill and Goldberg, 1968; Schacknow and Raab, 1973; Penner et al., 1974; Rabinowitz et al., 1976; Jesteadt et al., 1977). The previous study using Wang’s model (Deco and Rolls, 2006; Deco et al., 2007) did not show such a violation. However, stimulus intensity levels considered in the research (Deco and Rolls, 2006; Deco et al., 2007) reflected experimentally considered levels (Romo et al., 2004) and were limited in range.

The deviations from Weber’s Law in the low-intensity range were reported in many experiments. The magnitude of this effect was especially high in experimental studies on light brightness (Aubert, 1865; König and Brodhun, 1889; Blanchard, 1918; see Hecht, 1924; Von Helmholtz, 1866; Stiles and Crawford, 1933, 1934; Steinhardt, 1936; Blackwell, 1946; Aguilar and Stiles, 1954). In these studies (König and Brodhun, 1889; Blanchard, 1918; reported by Hecht, 1924), the highest reported values of Weber fractions were equal to 0.6–0.7 for the lowest light intensity levels and were 70-fold higher than Weber fractions in the medium to high light intensity ranges. Such magnitude of change of Weber fractions was not reported in the experiments on discrimination of pure tones (Riesz, 1928; McGill and Goldberg, 1968; Rabinowitz et al., 1976; Jesteadt et al., 1977). The experiment of Jesteadt, Wier, and Green involved pulse tones with the lowest intensity level of 5 dB in the presence of the 0 dB spectrum level noise. However, the authors also reported data for tones of intensity lower than 5 dB presented in the absence of noise. The Weber factions from these measurements were above the revealed relationship for intensities 5 dB and higher, and thus, could suggest a similar pattern of low-intensity violations as observed in the studies on light brightness. Although the violation of Weber’s Law in the low intensity range was also observed in the computational experiments presented here, its magnitude was much smaller. This suggested that some other neural mechanism could be involved in the violation of Weber’s Law for low intensities. Explanations linking this phenomenon to the quantum nature of light (Hecht, 1924), or retinal noise (Barlow, 1957) were previously investigated, but they were unlikely to explain the 70-fold difference in Weber fractions. The analysis of alternative neuronal mechanisms capable of producing such a magnitude of violation was beyond the scope of the current paper.

Our analysis predicted the violation of Weber’s Law in the high-intensity range. Such violation was observed in some experimental studies involving high stimulus intensities (König and Brodhun, 1889; Cobb, 1916; Holway and Pratt, 1936). However, this effect was debated in the scientific discourse, as it was not observed in many other studies. In particular, in the experiment of Jesteadt et al. (1977) the relation between stimulus intensities of pure tones and Weber fractions was decreasing. However, the study investigated the intensity range of 5–80 dB, far below the upper threshold of the human auditory system.

Theoretical research suggested the link between Weber’s Law and the scale invariance of RT distributions (Simen et al., 2016; Pardo-Vazquez et al., 2019). So far, the empirical evidence for the scale invariance was weak and did not cover the full range of stimulus intensity levels (Simen et al., 2016; Pardo-Vazquez et al., 2019). In this paper, we investigated the postulated scale invariance based on the modeled RT distributions. The modeled RT distributions showed scale invariant proximity but violated the property of scale invariance in the strict sense. The violation of strict scale invariance was not surprising considering finite-size effects and the constraints of neuronal networks. Such constraints were implemented into the considered model of the choice circuit and are also present in the biological networks. Our analysis suggested that the violation of scale invariance could be observed in RT skewness. Unlike mean and variance, the skewness was scale invariant. Our analysis showed systematic differences in the skewness of the modeled RT distributions. Such relation could be further investigated in empirical studies. Note that the estimates of RTs in behavioral studies involve non-decision time typically of app. 100 ms (which we did not assume here). Non-decision time affects skewness. Our analysis showed the proximity of the modeled RT distributions with the family of gamma distributions. Gamma distributions were useful in the context of scale invariance, as the scale invariance implied a constant value of the shape parameter, while differences in skewness implied different values of this parameter.

The theoretical investigations (Simen et al., 2016; Pardo-Vazquez et al., 2019) suggested that the emergence of Weber’s Law was noise-related. In such a view, the noise accompanying the evidence accumulation process was a function of inputs’ levels. This effectively rescaled the process leading to the invariance of Weber fractions. Conversely, the emergence of Weber’s Law in our view was linked to global inhibition producing a similar effect as divisive normalization (Heeger, 1992; Carandini and Heeger, 2012). Thus, the conceptual explanations were different. Further research is necessary to support either of these explanations.

Our analysis involved the linear relationship between stimulus intensity and its encoding in the stochastic spiking activity of neurons delivering the signal to the decision system. The relationship between stimulus and encoding was investigated in several experimental studies (e.g., Werner and Mountcastle, 1965; Mountcastle et al., 1969) and is one of the central topics in theoretical neuroscience (Dayan and Abbott, 2005, Gerstner et al., 2014). Evidence for the linear relationship came from the empirical studies using the dots motion discrimination tasks (Britten et al., 1993; Mazurek et al., 2003). The linear encoding scheme was also reported in the experiment on Weber’s Law using the vibrotactile frequency discrimination task (Romo et al., 2004). However, some studies (Scheler, 2017) showed that the spiking activity of a population of neurons (firing rate) had a lognormal distribution which suggested a logarithmic coding scheme. Neither our analysis nor the previous computational analyses (Deco and Rolls, 2006; Deco et al., 2007) excluded the possibility of the logarithmic encoding scheme as the alternative neuronal mechanism of the implementation of Weber’s Law in the brain.

Our model predicted that the convexity of the relation between stimulus intensities and the Weber fractions depended on the assumed choice probability, i.e., on how easy the discrimination was. Easy discrimination was linked to stronger convexity. This prediction can be tested in empirical research and could help to experimentally verify the mechanism behind Weber’s Law. It is unplausible that such an effect could be related to a mechanism that is based on stimulus encoding.

The current study has several limitations. Our analyses did not take into account realistic latencies of neurons delivering the signal to the choice circuit. Such latencies could produce non-linearity in stimulus encoding. I have conducted a separate set of model simulations implementing such latencies. The differences between Weber fractions from simulations including and excluding latencies were not statistically significant.

Note also that the parametric approximation of modeled RT distributions should be considered with caution. The modeled RT distributions depended on the attractor dynamics as well as the underlying update process. The underlying update process was assumed to follow the Poisson point process except for a constant update delay after emitting a spike. Although modeling spikes with the Poisson process were motivated by theoretical considerations and previous modeling practice (Dayan and Abbott, 2005; Wójcik, 2018), alternative processes could be considered. Understanding the impact of the chosen process on the parametric fit of RT distributions would require using a different model specification and was not analyzed in this paper.

## Data Availability

The model was implemented in R (R Core Team, 2021) version 4.1.1 (2021-08-10). Code and datasets obtained during the current study are available in the Github repository, https://github.com/MarcinPenconek/Weber-s-Law-as-the-Emergent-Phenomenon.
